# A Rice Ancestral Genetic Resource Conferring Ideal Plant Shapes for Vegetative Growth and Weed Suppression

**DOI:** 10.3389/fpls.2021.748531

**Published:** 2021-11-26

**Authors:** Noritoshi Inagaki, Hidenori Asami, Hideyuki Hirabayashi, Akira Uchino, Toshiyuki Imaizumi, Ken Ishimaru

**Affiliations:** ^1^Research Center for Advanced Analysis, National Agriculture and Food Research Organization (NARO), Tsukuba, Japan; ^2^Western Region Agricultural Research Center (Kinki, Chugoku, and Shikoku Regions), National Agriculture and Food Research Organization (NARO), Fukuyama, Japan; ^3^Institute for Plant Protection, National Agriculture and Food Research Organization (NARO), Tsukuba, Japan; ^4^Institute of Crop Science, National Agriculture and Food Research Organization (NARO), Tsukuba, Japan; ^5^Central Region Agricultural Research Center (Kanto, Tokai, and Hokuriku Regions), National Agriculture and Food Research Organization (NARO), Tsu, Japan

**Keywords:** ancestral genetic resource, Asian cultivated rice (*Oryza sativa* L), light reception efficiency, plant shape, tiller angle, vegetative growth, weed control, wild rice (*Oryza rufipogon* Griff.)

## Abstract

To maximize crop growth, crops need to capture sunlight efficiently. This property is primarily influenced by the shape of the crops such as the angle, area, and arrangement of leaves. We constructed a rice (*Oryza sativa* L.) inbred line that displayed an ideal transition of plant shapes in terms of sunlight receiving efficiency. During vegetative growth, this line exhibited tiller spreading with increased tiller number, which formed a parabolic antenna-like structure. The architecture probably improved light reception efficiency of individuals compared with the recurrent parent. The line achieved not only acceleration of the vegetative growth, but also significant suppression of weed growth under the canopy. The increased light reception efficiency of the line has consequently reduced the amount of incident light to the ground and supplied significant competitiveness against weeds. The spread tillers became erect from the entry of the reproductive growth phase, adaptively sustaining light reception efficiency in thicker stands. The line carries a small chromosomal segment from *Oryza rufipogon* Griff., a putative progenitor of Asian cultivated rice. The introduced chromosome segment had little effect on grain yield and quality. Our results shed light on potentials hidden in the wild rice chromosome segment to achieve the valuable traits.

## Introduction

Asian cultivated rice, *Oryza sativa* (*O. sativa*), is one of the world’s most important crops, sustaining several billion people as a staple food (Global Rice Science Partnership, 2013). To ensure permanence of humanity, improvement of rice cultivation systems should be required not only from economic perspectives, but also from viewpoints based on sustainability.

*O. sativa* was domesticated from a wild rice species, *Oryza rufipogon* (*O. rufipogon*), which is distributed throughout Asia and Oceania (Chen et al., 2019). Human ancestors empirically selected beneficial lines along their cropping systems from the progenitor. Such domestication processes substantially narrowed the genetic diversity at genetic bottlenecks formed during these winnowing steps (Doebley et al., 2006). Comprehensive genome sequencing and analyses of *O. rufipogon* and *O. sativa* quantitatively ascertained the vast reduction in genetic diversity that occurred during domestication (Zhu et al., 2007; Huang et al., 2012; Chen et al., 2019). On the other hand, modern breeding has certainly attained some success in constructing elite cultivars using the limited amount of genetic variation still retained in cultivated rice plants and acquired mutations in key genes involved in critical agricultural traits; however, this strategy has been sometimes confronted with limits attributed to a low level of genetic variation. Therefore, the positive resurrection of genetic variations lost during domestication will be a powerful tool to solve deadlocks in genetic improvement (Kamboj et al., 2020).

During the rice domestication, one of the most drastic events was exclusion of prostrate plants, mostly exhibited by wild rice, which triggered to form majority of the erect plants found in almost all the modern rice cultivars. This selection probably preceded at the early phase of the domestication, which conferred an increase in yield per area by supplying the ability for dense planting. This characteristic was extremely important in the ancient times when farmland expanding ability was low and/or farmland with appropriate conditions was restricted.

In 2008, the domestication locus responsible for the erect tiller trait was identified and named the *Prostrate growth 1* (*Prog1*), a gene located on the short arm of chromosome 7 (Jin et al., 2008; Tan et al., 2008). The *Prog1* gene encodes a C_2_H_2_-type zinc finger transcription factor (Agarwal et al., 2007). Recently, Wu et al. (2018) reported that *O. rufipogon* (accession DXCWR) possesses approximately 110 kbp of an additional chromosomal segment named the *RICE PLANT ARCHITECTURE DOMESTICATION* (*RPAD*) in the near vicinity of the *Prog1* gene. The *RPAD* segment contains seven tandem *Prog1*-like genes; at least three of the *Prog1*-like genes were confirmed to modify plant architecture. The erect habit of cultivated rice plants was presumed to be guided by the synergistic effects of sequence changes in the *Prog1* and deletion of the *RPAD* segment from the *O. rufipogon* genome (Huang et al., 2020).

Growth of crops, including rice plants, certainly requires the adequate sunlight as a basal energy source for photosynthesis and is greatly affected by the efficiency of sunlight reception. In fact, Monteith (1977) experimentally found that primary crop production is proportional to the amount of intercepted light energy. Therefore, improving the light-intercepting characteristic of plant shape is expected to promote crop growth. The exact plant shape, which is most effective in perceiving solar radiation, is a point of debate. Theoretical studies examining the relationship between leaf angles and dry matter production have suggested that erect leaves are preferable for increasing the capture of sunlight and enhancing photosynthetic production during the late vegetative growth phase when leaves grow thicker and become mutually shaded (Monteith, 1965; Duncan et al., 1967). In terms of growth promotion during the late vegetative phase, rice lines possessing erect leaves and/or panicles have been constructed that can achieve some increase in yield (Sakamoto et al., 2006; San et al., 2018; Fei et al., 2019). In contrast, Monteith (1965) and Duncan et al. (1967) also reported that inclined leaves are more efficient in perceiving light when there is no mutual shading as typically found in plants with a low leaf area index (LAI), implying that a spreading phenotype is preferable for plants during the early vegetative growth phase. These interpretations indicate that the ideal plant shape for efficient light capture changes depending on the growth phase of the crop.

Among biotic factors interfering with crop yield, weeds constantly invade and spread in fields and are a major cause of reduced yield in all the regions of the globe (Chauhan, 2020). Conventional weed control by manually removing weeds from the field is burdensome and the frequent application of herbicides is expensive and comes with ecological costs such as environmental destruction risk and/or the appearance of herbicide-resistant weeds. Fewer herbicide applications relieve not only the economic burden, but also the ecological issues; however, these applications alone will reduce yields of crops due to insufficient weed control. More ecological approaches, which can substantially reduce herbicide applications, i.e., construction of cultivars with an appropriate plant shape that preserves strong competitiveness against weeds, have been considered (Johnson et al., 1998; Fischer et al., 2001; Dass et al., 2017).

The ideal plant shape for rice to improve weed competitiveness is a plant with many tillers and sloping leaves (Gibson et al., 2003; Koarai and Morita, 2003). These traits would form a deep canopy, limit light penetration onto the ground, and then suppress the growth of weeds under the canopy. From these perspectives, researchers have sought and examined cultivars displaying such ideal plant shapes for strong weed competitiveness. However, it was feared that such traits would lead to a severe decrease in yield due to energy loss by mutual shading of the crop leaves, i.e., the features conflict with the preferable plant shape for high yields in the late vegetative phase. Therefore, cultivars with compatible plant shape are earnestly demanded, especially in developing countries with little economic capacity, which would reduce the weeding costs. Even in developed countries, the decrease of herbicide application has great merits to relieve not only environmental loads, but also appearance of herbicide-resistant weeds (Annett et al., 2014; Heap and Duke, 2018; Islam et al., 2018).

From the perspective of agricultural management, sparse planting of rice seedlings is demanded to save labor and the production cost. However, the sparse planting brings to a new weakness in weed control because the canopy closure of rice plants, which represses weed growth, is delayed. We have to examine ideal plant shapes for the sparse planting that maintain yield and are compatible with weed control.

In this study, we established the rice near-isogenic line (NIL) of Koshihikari holding ideal plant shapes that are compatible with yield and weed control even under sparse planting condition. Koshihikari is the most widely accepted *japonica* cultivar for staple food in Japan due to its good taste and is recognized to be a monumental existence that has made great contributions to rice breeding in Japan as a mating parent. Koshihikari has erect tillers, which characteristic is generally found in common cultivated rice. The Koshihikari-background NIL contains a genomic segment of chromosome 7 from an accession of *O. rufipogon* collected in Thailand, which segment carries the novel *Prog1* sequence with the *RPAD*. The NIL displayed the spreading tiller phenotype in the vegetative growth phase; however, the tillers began to erect from the entry of the reproductive growth phase. This transition between the plant shapes complies with maintenance of optimal light receiving efficiency throughout plant development; therefore, the NIL balances promoted growth and significant weed suppression. These results suggest that the ancestral genetic resources used in this study have a great ability to upgrade current rice farming to innovative systems fitting with the United Nations Sustainable Development Goals (SDGs)^1^.

## Materials and Methods

### Plant Materials and Field Growth Conditions

In this study, Koshihikari, a cultivar of *japonica* rice (*O. sativa*), was used as the control. We used GP9-7, the NIL for Koshihikari, containing a segment of chromosome 7 from an accession of *O. rufipogon* collected in Thailand (IRGC Acc. No. 104814). This line was selected by marker-assisted breeding from lines obtained in the BC_4_F_2_ generation of KRIL31 backcrossed to Koshihikari. KRIL31 is a line in the chromosome segment substitution lines (CSSLs) containing chromosomal segments from wild relatives in the background of *japonica* cultivars, which has been constructed in our former work (Hirabayashi et al., 2010). This study was conducted at the Tsukuba-Kannondai test fields for NARO (N-36.0°, E-140.1°, 22 m above sea level) and at the Western Region Agricultural Research Center, Fukuyama test fields for NARO (N-34.5°, E-133.4°, 1 m above sea level). Details of the field conditions in our experiments were described in the text or the legends of the figures. A summary of climate conditions in the years of the field experiments indicates in Supplementary Table 1.

### Cross-Fertilization and Genotyping

After emasculation of pollen on female plants by soaking panicles in hot water at 43°C for 7 min, mating was accomplished by sprinkling the pollen of male plants onto emasculated female stigmata. DNA was extracted from a small piece of a leaf tip using the DNeasy Plant Mini Kit (QIAGEN, Hilden, Germany) following the instruction of the manufacturer. Genotyping was carried out by PCR using the single sequence repeat (SSR) markers (Temnykh et al., 2000; McCouch et al., 2002; International Rice Genome Sequencing Project, 2005) listed in Supplementary Table 2. The PCR mixture (10 μl) consisted of 0.5 μl of template DNA, 5 μl of GoTaq Green Master Mix (Promega, Madison, WI, United States), and 0.6 μl of 10 μM primers. Amplification was performed for 35 cycles of 94°C (30 s), 55°C (30 s), and 72°C (30 s). Amplified DNA products were electrophoresed in 3.0% (w/v) NuSieve 3:1 Agarose Gels (Lonza, Basel, Switzerland, United Kingdom).

### Growth Analyses

At designed intervals, above ground parts of randomly selected seven plants grown in a paddy field (500 m^2^; interplant space = 18 cm × 25 cm; 22.2 plants m^–2^) were harvested. Leaf area was measured with a leaf area meter (AAM-9, Hayashi Denko Corporation Ltd., Tokyo, Japan) and LAI was calculated. All the samples were oven dried for 2 days prior to measuring dry weight. The crop growth rate (CGR) (g m^–2^ day^–1^) and net assimilation rate (NAR) (g m^–2^ day^–1^) were calculated using the following equations:


(1) C⁢G⁢R=K⁢(W⁢2-W⁢1)/(t⁢2-t⁢1)

(2) N⁢A⁢R=(W⁢2-W⁢1)/(t⁢2-t⁢1) {(l⁢n⁢(A⁢2)-l⁢n⁢(A⁢1))/(t⁢2-t⁢1)}

where, *K* indicates the plant density (plants m^–2^), *W1* and *W2* represent the above ground dry weight (g) at times *t*1 and *t*2, respectively, and *A*1 and *A*2 represent the leaf area (m^2^) at *t*1 and *t*2, respectively.

### Measurement of Tiller Inclination Angle and Vegetation Cover Rate

Tiller inclination angles were measured as the angle from the horizontal to a tiller located on the outermost circumference. The vegetation cover rates were calculated from binarized images of plants taken from directly above as the ratio of pixels corresponding to the plant body to the total number of pixels. The binarized images were generated from the a^∗^ signals in the CIELAB (Commission internationale de l’éclairage L^∗^ a^∗^ b^∗^) color space of the original images converted with the ImageJ software version 1.52 k^2^ with a color space converter plugin (LPX color; LPIXEL, Tokyo, Japan).

### Assays of Weed Growth in Competition With Rice

In this study, *Echinochloa crus-galli* (L.) Beauv. var. *formosensis* Ohwi (*E. crus-galli* var. *formosensis*), a well-known grass weed in Japan, was used. At 10 days after planting (DAP) of rice seedlings on paddy fields (interplant space = 30 cm × 30 cm; 11.1 plants m^–2^), *E*. *crus-galli* var. *formosensis* at the first leaf stage was transplanted to the middle of rice rows. The number of tillers of *E*. *crus-galli* var. *formosensis* was measured every other week. A total of 10 weed plants per plot (1.8 m × 3.3 m = 5.94 m^2^) were examined in three repetitions. In addition, all the *E*. *crus-galli* var. *formosensis* naturally emerging in the three plots of the field were sampled 10 days before rice harvest and the number of emerging weeds, their tiller number, and their dry matter weight were measured.

### Measurements of the Relative Photosynthetic Photon Flux Density

Vertical transitions of the RPPFD in the rice stand were measured on a cloudy day at noon using a quantum sensor (LI-190SB, LI-COR, Lincoln, NE, United States). Simultaneous measurements were carried out for locations every 10 cm from the ground surface in the rice stand and above the canopy (1 m from the ground surface) using the measurements taken above the stand as 100%. The measurements were recorded three times and the mean value was used in the analysis.

Transitions of the RPPFD in the rice stand during the growth were measured using a line quantum sensor (366813M; Ollie Corporation Ltd., Osaka, Japan). Simultaneous measurements were carried out in the rice stand (ground surface) and outside the stand (1 m from the ground surface) using measurements taken outside the stand as 100%. Measurements were recorded once a week from transplanting to harvest. The measurements were recorded three times and the mean value was used in the analysis. At the same time, the number of rice tillers was measured for three plots that consist of 10 plants.

### Grain Yield Measurement and Eating Quality Tests

A total of 12 individual plants randomly selected from 100 plants grown in a field (4 m × 3.3 m = 13.2 m^2^; interplant space = 36 cm × 36 cm; 7.7 plants m^–2^) were harvested as one plot. The panicles in each plot (three plot replicates) were mechanically threshed, the obtained grains were dried, and the weight of the paddy rice was measured to determine grain yield. Samples for the taste test were obtained from plants growing in the same field as those used for grain yield measurements. Taste tests were conducted at the AiHO Rice Cooking Research Institute (Toyokawa, Aichi, Japan). Protein and amylose contents were measured using a Rice Composition Analyzer (Shizuoka Seiki Corporation Ltd., Fukuroi, Japan). Mido Meter (Toyo Rice, Wakayama, Japan) and Rice Taste Analyzer (Satake Corporation, Higashi-Hiroshima, Japan) were used for objective comparisons of taste-related factors. Values for texture of cooked rice were examined by a Tensipresser (Takemoto Electric Incorporation, Tokyo, Japan).

### Statistical Analysis

Statistical analyses were conducted using the statistical computing software R, version 3.6.3^3^.

## Results and Discussion

### Introductory Description of the Near-Isogenic Line (GP9-7)

**FIGURE 1:**
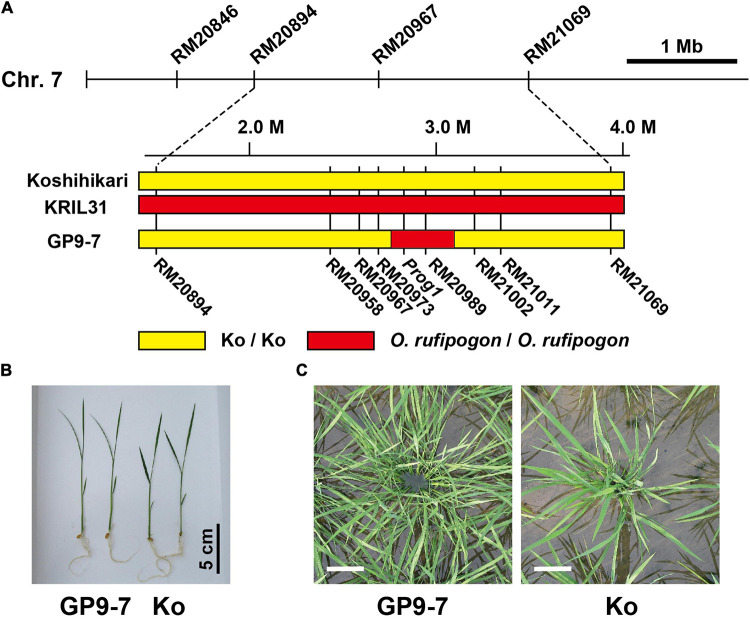
Basic properties of GP9-7. **(A)** A comparative diagram showing the genotyping results of GP9-7, KRIL31, and Koshihikari. The yellow zones indicate the segments derived from Koshihikari and the red zones represent the segments derived from *Oryza rufipogon* (*O. rufipogon*). **(B,C)** Typical plant shapes of the rice plants are used in this study. The photograph **(B)** shows typical nursling seedlings of Koshihikari and GP9-7 at 10 days after sowing. The photographs **(C)** indicate typical plant shapes in paddy fields taken from directly above the plants at 33 days after planting (DAP). Scale bars: 10 cm.

For the first trial, we examined the agricultural traits of KRILs (Hirabayashi et al., 2010) in field conditions in which seedlings were planted in sparse (interplant space = 36 cm; 7.7 plants m^–2^) or dense (interplant space = 18 cm; 30.9 plants m^–2^) plant densities. The KRILs consist of 40 lines, each of which possess one or few chromosomal segments of *O. rufipogon* (IRGC accession number 104814) in Koshihikari background. This CSSL (KRILs) were constructed to examine whether the wild rice genetic resources can be used to overcome weaknesses and/or add new features to Koshihikari. Among the KRILs, KRIL31 displayed wide spreading with an increased number of tillers, especially in the sparsely planted condition (Supplementary Figure 1). Therefore, we focused on these traits that altered vegetative growth presumably caused by a change in their light-receiving posture, which could also provide strong weed competitiveness. Interestingly, KRIL31 did not have a clear dwarf phenotype, even though its tiller number had more than doubled compared with that of Koshihikari. The BC_1_F_1_ plants derived from a cross between KRIL31 and Koshihikari showed both the traits, indicating these traits were inherited dominantly (data not shown). KRIL31 possesses almost all of the long arm of chromosome 9 and three small segments in chromosomes 1, 3, and 7 from *O. rufipogon* (Hirabayashi et al., 2010; Supplementary Figure 2A). KRIL31 tillers maintained a strong spreading trait throughout plant development. When the chromosome 9 segment from *O. rufipogon* replaced that of Koshihikari, the spreading trait in the reproductive phase disappeared (data not shown). Yu et al. (2007) indicated that the *Tiller Angle Control 1* (*TAC1*) gene, which mediates tiller angle during the reproductive stage (late compact stage), is localized in the long arm of chromosome 9. This study aligns with our observation that when this segment was replaced with Koshihikari, resultant progenies lost tiller inclination during the reproductive growth phase. In contrast, the segment of chromosome 7 from *O. rufipogon* was linked to leaning tillers only during the vegetative growth phase. To define the segment responsible for the spreading of tillers, we conducted phenotypic and genotypic analyses of 430 lines of the BC_2_F_2_ generation, which indicated that the loci for spreading and increasing tiller number were detected between two SSR markers, RM20967 and RM20999 (Supplementary Figure 2B). We selected the NIL, GP9-7 from 300 lines of the BC_3_F_4_ generation by genotypic analyses based on the above information. The chromosomes of GP9-7 had almost reverted to Koshihikari form except for the region between two SSR markers, RM20973 and RM21002 (Figure 1A). We used this line in all the subsequent studies.

### Inserted Chromosomal Segment of GP9-7

As mentioned above, GP9-7 contains a small segment of chromosome 7 from *O. rufipogon* (IRGC accession number 104814) between two SSR markers, RM20973 and RM21002 (Figure 1A). The segment between the two markers corresponds to 358 kbp based on the Nipponbare genome sequence (International Rice Genome Sequencing Project, 2005). This segment includes the *Prog1* gene that is involved in key processes required for domestication and has been reported to determine tiller inclination angle and number of tillers (Jin et al., 2008; Tan et al., 2008). The *Prog1* gene encodes a single C_2_H_2_ zinc-finger transcription factor (Agarwal et al., 2007; Supplementary Figure 3). The *Prog1* sequences of *O. rufipogon* collected in China have been sequenced comprehensively, but the *Prog1* sequence of GP9-7, which is from an accession of *O. rufipogon* (IRGC accession number 1048141) collected in Thailand, is novel. This sequence is similar to that of cultivated rice with a deletion of 9 bp in the open reading frame that causes a deletion of three residues near an EAR (ethylene-responsive element binding factor-associated amphiphilic repression)-like motif (Kagale et al., 2010; Supplementary Figure 3). Wu et al. (2018) proposed that the *RPAD* region is also involved in determining plant shape, located on the vicinity of the *Prog1* gene in the *O. rufipogon* genome. To verify the insertion of the *RPAD* segment in GP9-7, a PCR assay was performed using a primer set to detect the *RPAD* insertion. The assay revealed that GP9-7 probably possesses the *RPAD* segment (Supplementary Figure 4) that is likely to confer the ability to alter plant shape together with the *Prog1* gene.

### Tiller Inclination and Tiller Number in GP9-7

Nursling seedlings of GP9-7 were slightly thinner and more elongated compared with Koshihikari (Figure 1B). After planting on paddy fields in sparse condition (interplant space = 36 cm × 36 cm; 7.7 plants m^–2^), the number of GP9-7 tillers increased and began leaning more each day (Supplementary Figure 5; Supplementary Movie 1), comparable with previously reported inbred lines containing similar chromosomal segments of *O. rufipogon* (Jin et al., 2008; Tan et al., 2008). The number of GP9-7 tillers was roughly equivalent to those of Koshihikari until about 15 DAP; however, afterward, the number of GP9-7 tillers increased significantly, reaching more than three times those of Koshihikari by 43 DAP (Supplementary Figure 5). The plastochron of GP9-7 was almost equivalent to that of Koshihikari (data not shown). This observation and the biased increase in tiller number toward the later developmental stages suggested that higher order tillers that normally do not appear in Koshihikari did emerge from GP9-7. Tillers that elongate too much can lead to mutual shading, but the emerging GP9-7 tillers spread in all the directions with very few overlaps and then assumed a parabolic antenna-like structure (Figure 1C). Therefore, leaves on the tillers of GP9-7 did not compete with each other for light.

**FIGURE 2:**
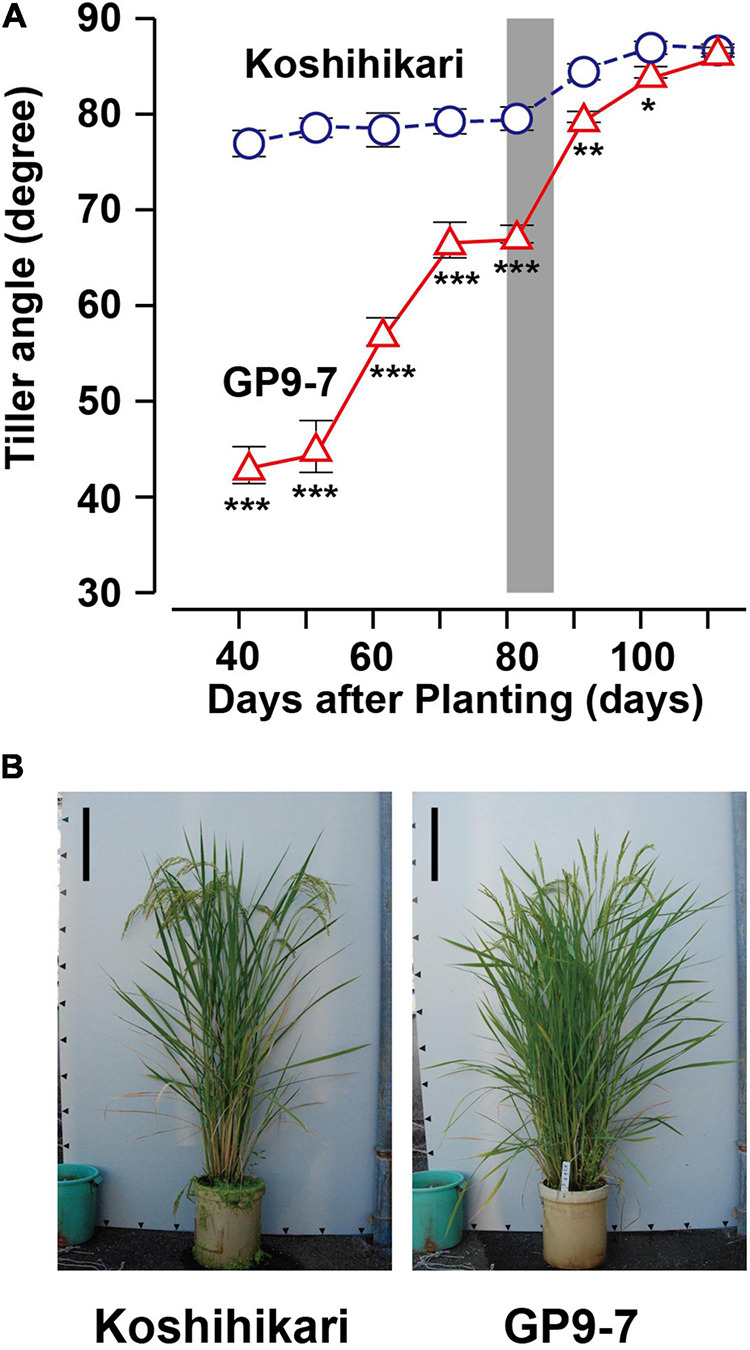
Basic properties of GP9-7 toward the reproductive growth phase. **(A)** Developmental transitions of tiller angles from horizontal in Koshihikari (blue circles with broken lines) and GP9-7 (red triangles with solid lines) grown in a test field at Tsukuba, Japan in the summer of 2020 (planting date: May 21, 2020). The tiller angles of the same six plants were continuously measured and the mean values with SE were shown in the graph. The period for heading is indicated by a vertical gray bar. Symbols, ***, **, and * indicate statistically significant differences compared to Koshihikari at *P* < 0.001, *P* < 0.01, and *P* < 0.05, respectively, calculated by the Welch’s *t*-test. The photographs **(B)** display typical plant shapes of Koshihikari and GP9-7 during the ripening phase (25 days after heading). Scale bars: 20 cm.

The erect tiller trait in cultivated rice, including Koshihikari, is caused by the upward curving of laterally emerging non-elongated internodes (Supplementary Figure 6A). Tan et al. (2008) reported that the curved internodes are due to their asymmetric development in which the near-ground border, the outermost cell layer of the tiller base closest to the ground, is longer than the border further away from the ground. Cell sizes for both the borders were almost equivalent suggesting that the curved internodes are due to an increase in cell number on the near-ground border. In contrast, the laterally emerging non-elongated internodes of GP9-7 were straight (Supplementary Figure 6A), probably due to symmetric development between the near-ground and the far-ground borders. This observation does not mean that GP9-7 is not gravitropic. Coleoptiles of dark-germinated Koshihikari and GP9-7 seedlings were clearly gravitropic in an experiment in which seedlings were rotated horizontally at the midpoint of an incubation period (Supplementary Figure 6B). Furthermore, tillers of GP9-7 began to rise during the transition from the vegetative to the reproductive growth phase (Figure 2A; Supplementary Movie 2). This trait was derived from a clear gravitropic bend in GP9-7 nodes, especially the node between internodes III and IV (Supplementary Figure 6C), resulting in erect panicles (Figure 2B). This trait favorably increasing the light reception efficiency in the thicker stand as is often the case in the reproductive growth phase (Sakamoto et al., 2006; San et al., 2018; Fei et al., 2019). In addition, this trait is preferable for modern agricultural harvesting methods using combine harvesters. As mentioned above, GP9-7 exhibited a clear transition in plant shape from the vegetative growth phase during which plants had inclined tillers to the reproductive growth phase when the tillers were erect.

### Accelerated Growth of GP9-7

**FIGURE 3:**
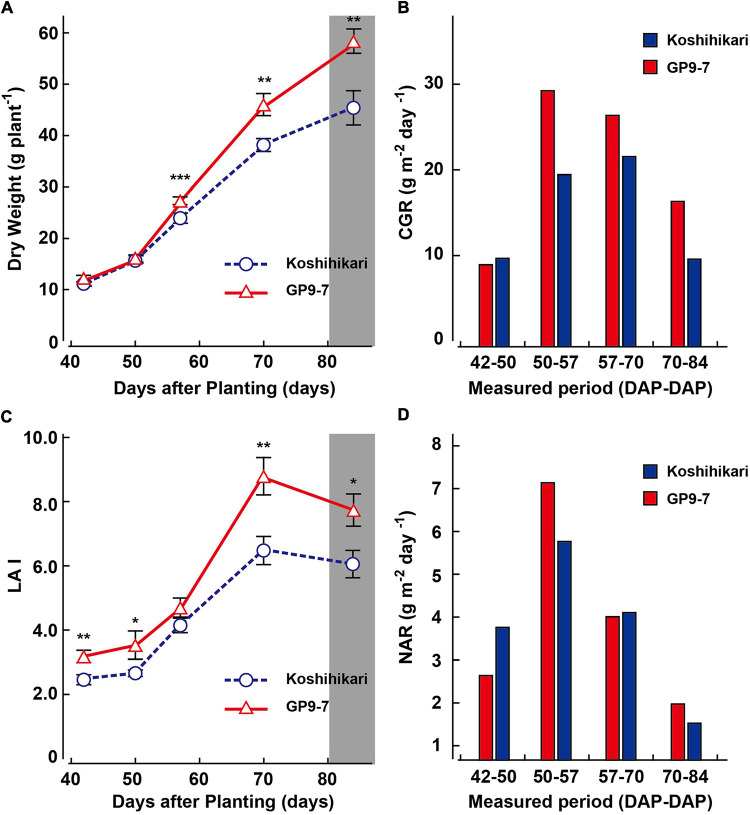
Developmental transitions of growth traits of Koshihikari (blue circles with broken lines or blue bars) and GP9-7 (red triangles with solid lines or red bars) from the vegetative to the reproductive growth phase. Transitions of dry weight per plant **(A)**, crop growth rate (CGR) **(B)**, leaf area index (LAI) **(C)**, and net assimilation rate (NAR) **(D)** of Koshihikari and GP9-7 grown in a test field at Tsukuba, Japan in the summer of 2018 (planting date: May 9, 2018). Mean values of the dry weights and leaf area indexes obtained from seven plants are plotted. The SE values are shown as error bars in the graphs. The period for heading is indicated by a vertical gray bar. Symbols, ***, **, and * indicate statistically significant differences compared to Koshihikari at *P* < 0.001, *P* < 0.01, and *P* < 0.05, respectively, calculated by the Welch’s *t*-test.

GP9-7 and Koshihikari were grown in a field in Tsukuba, a region in eastern Japan, using local agricultural practices (interplant space = 18 cm × 25 cm; 30.9 plants m^–2^). Leaf area and shoot dry matter weight were measured at intervals; LAI, CGR, and NAR were calculated from these values (Figure 3). Early vegetative growth of GP9-7 up to 50 DAP was indistinguishable from Koshihikari in terms of dry matter weight per plant; however, beginning at 57 DAP, the increase in dry matter weight of GP9-7 was significantly accelerated (Figure 3A). Therefore, the CGR values for GP9-7 during these periods were higher than those of Koshihikari (Figure 3B). Since the CGR is recognized as the product of LAI and NAR, it is easy to distinguish which factor contributes more to the increase in the CGR. The factor that promoted the CGR of GP9-7 from 50 to 57 DAP was attributed to a temporary increase in the NAR during the same period (Figures 3B,D). In contrast, the higher CGR in the subsequent period (57 to 85 DAP) was due to an increase in LAI (Figures 3B,C). The temporary increase in the NAR may be due to improved light-intercepting characteristics in the stand rather than from an increase in the photosynthetic activity of leaves, as discussed in more detail in the next section.

### Light Intercepting Ability of GP9-7

**FIGURE 4:**
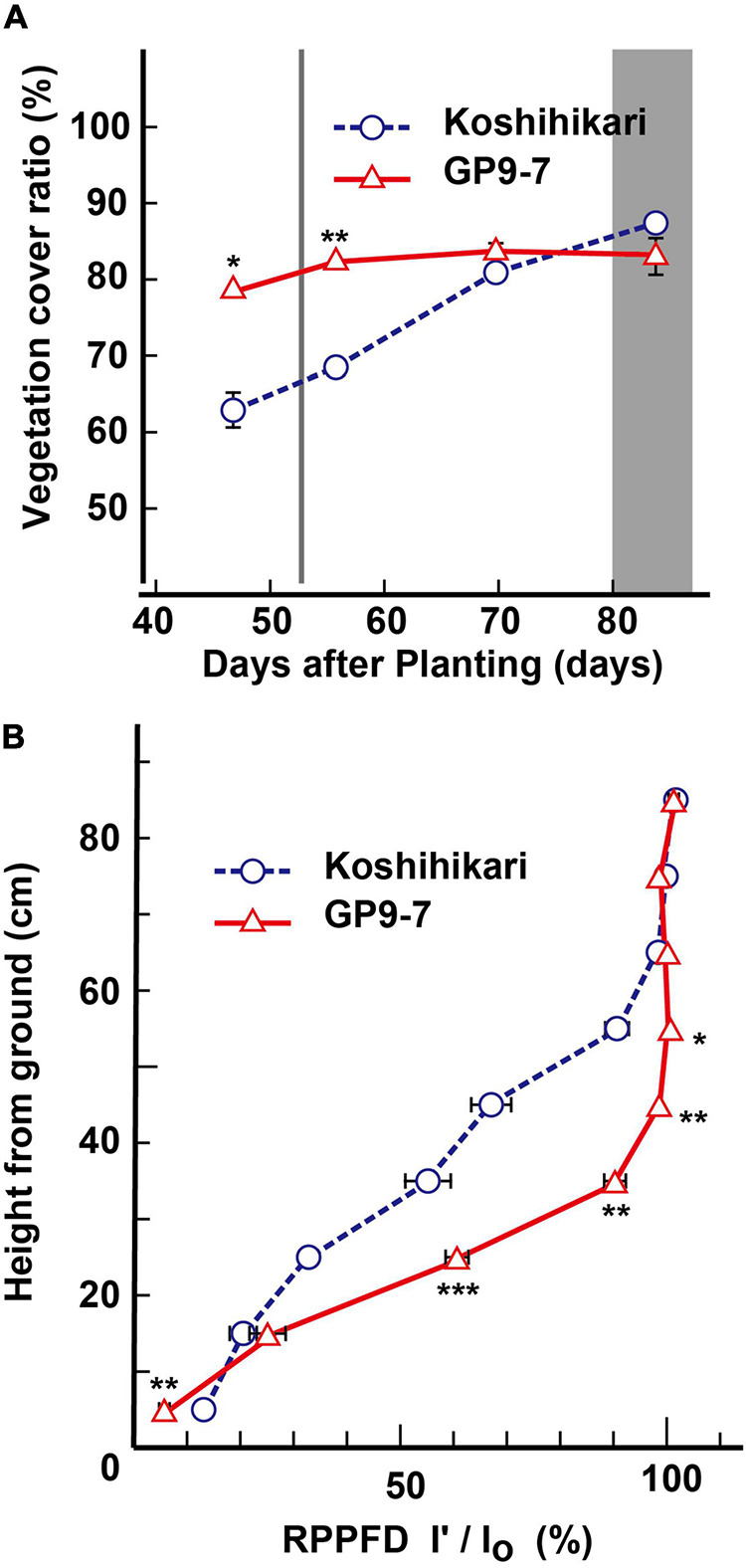
Light reception-related properties of GP9-7. **(A)** Developmental transitions in vegetative coverage of Koshihikari and GP9-7 in the test field at Tsukuba, Japan in the summer of 2018 (planting date: May 28, 2018). The period for heading is indicated by a vertical gray bar. This analysis was performed according to Materials and Methods, using images continuously taken at the same three points in the field, and the mean values with SE were indicated in the graph. **(B)** Vertical transitions of the relative photosynthetic photon flux density (RPPFD) of Koshihikari and GP9-7 measured on the 52nd DAP that is shown by a vertical gray line in panel **(A)**. Symbols, ***, **, and * indicate statistically significant differences compared to Koshihikari at *P* < 0.001, *P* < 0.01, and *P* < 0.05, respectively, calculated by the Welch’s *t*-test. Values for Koshihikari are indicated with blue circles with broken lines and values for GP9-7 are represented with red triangles with solid lines.

The increased growth performance of GP9-7 is probably due to the enhanced amount of light energy received by the individuals attributed to their distinctive shape. Tiller emergence of GP9-7 plants was greater than that of Koshihikari and the tillers were arranged radially causing less mutual shielding (Figure 1C; Supplementary Figure 5). Therefore, the vegetation cover ratios of GP9-7 in the midvegetative phase were significantly higher than those of Koshihikari (Figure 4A). This result suggests that the light-intercepting efficiency of GP9-7 was increased by the plant shape. The RPPFD of GP9-7 in the stand at 53 DAP had a characteristic vertical profile (Figure 4B) when the NAR of GP9-7 was at its peak (Figure 3D). The vertical RPPFD profile in cultivated rice plants with erect tillers usually indicates a gradual decrease in light intensity from the canopy to the ground surface. In alignment with this expectation, the vertical RPPFD of Koshihikari gradually decreased below 60 cm (Figure 4B). In contrast, an extreme decrease in the RPPFD was detected below 40 cm in GP9-7 (Figure 4B). This characteristic was attributed to the parabolic antenna-like morphology of GP9-7 that had many uniformly spread tillers with a constant slope, forming wide shadows without mutual shielding (Figure 1C). Under the condition, slightly excessive LAI should be connected to effective light capture. In addition, tilted leaf sheaths of GP9-7 were fully exposed to the sky (Figure 1C), which implies that photosynthetic assimilation in the leaf sheaths contributes growth promotion of GP9-7. Actually, Guo et al. (2011) reported that rice leaf sheaths had active photosynthetic apparatus such as leaf blades. Moreover, the leaf sheath photosynthesis was accounted for 10 to 20% of the final yield. Our observation that leaf sheaths of GP9-7 were dark green, such as active photosynthetic organs, is consistent with these interpretations. Thus, efficient light receiving of GP9-7 attributed to the distinctive plant shape during the period from 50 to 57 DAP that could be associated with the temporary improvement of the NAR during this period (Figure 3D). In the subsequent period (57 to 85 DAP), GP9-7 had the extremely higher LAI, which raises the risk of mutual shielding as an inevitable consequence. However, the plant shape transition of GP9-7 from 57 DAP, when tillers began to erect (Figure 2A), could adaptively sustain a preferred light receiving character in the thicker stand.

### Weed Control in GP9-7 Stand

**FIGURE 5:**
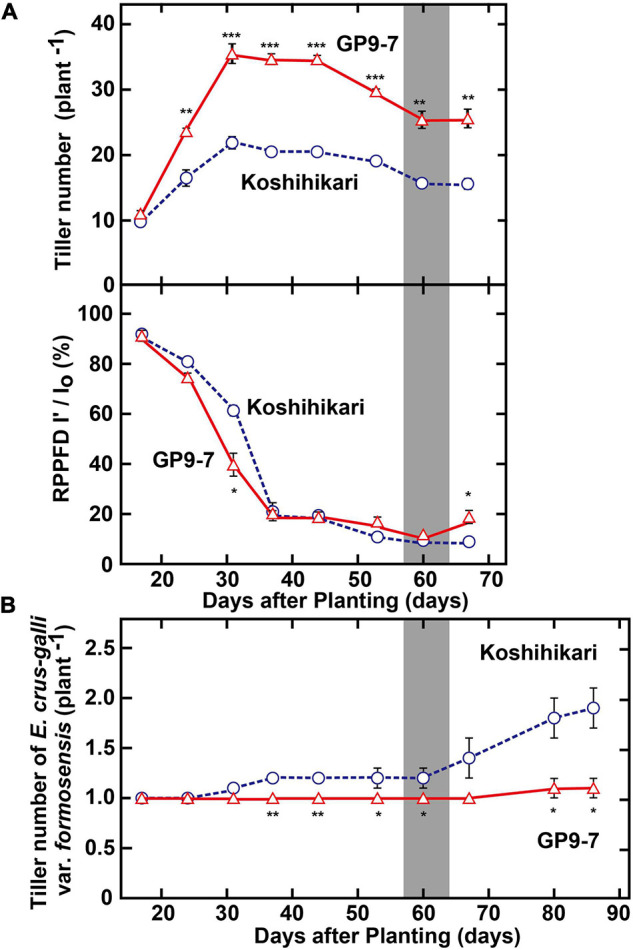
Weed suppression-related properties of GP9-7. **(A)** Developmental transitions in tiller numbers and the RPPFD at ground level of Koshihikari and the GP9-7 in the test field in Fukuyama, Japan in the summer of 2019 (planting date: June 15, 2019). The periods for heading are indicated by vertical gray bars. **(B)** Developmental transitions in tiller numbers of the transplanted weed, *E. crus-galli* var. *formosensis* under the canopy formed by rice stands. Symbols, ***, **, and * indicate statistically significant differences compared to Koshihikari at *P* < 0.001, *P* < 0.01, and *P* < 0.05, respectively, calculated by the Student’s *t*-test. Values for Koshihikari are indicated with blue circles with broken lines and values for GP9-7 are represented with red triangles with solid lines.

**FIGURE 6:**
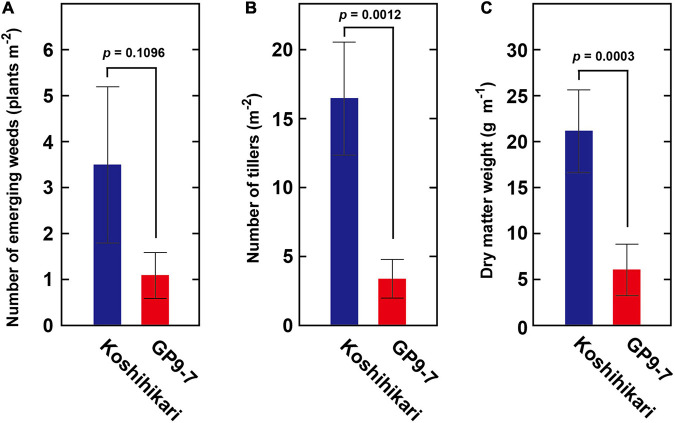
The weed competitiveness of GP9-7 was evaluated by measuring the growth of naturally occurring weeds in the summer of 2019 (planting date: June 15, 2019). The number of emerging weeds **(A)**, their tiller number **(B)**, and the dry matter weight **(C)** of naturally occurring *E*. *crus-galli* var. *formosensis* under the canopies of Koshihikari or GP9-7 are indicated. Mean values obtained from three plots for Koshihikari and GP9-7 with the SE values are indicated with blue and red bars, respectively. The *p*-values were calculated by the Student’s *t*-test and are reported above the bar graphs.

Ground coverage proceeded more rapidly in GP9-7 than in Koshihikari (Figure 4A), a factor that restricted light penetration onto the ground surface. Furthermore, the RPPFD just above the ground surface under the GP9-7 canopy at 53 DAP was 5% of that above the canopy and significantly lower than that of Koshihikari (Figure 4B). Since the lower ground level RPPFD is preferable for optimal weed control (Gibson et al., 2003; Koarai and Morita, 2003), the RPPFD of the ground level in GP9-7 stands was examined in detail in Fukuyama, a region in western Japan. In this study, the planting date was mid-June (June 15, 2019), about a month later than that at Tsukuba (mid-May). Although the heading date relative to the DAP shown in Figure 5 was significantly different from experiments conducted in Tsukuba (Figures 2–4), the progression of vegetative growth was essentially similar to that of Tsukuba-grown plants. After planting, the number of GP9-7 tillers increased (Figure 5A) and spread uniformly, significantly decreasing RPPFD of the ground level at approximately 30 DAP (Figure 5A). After 35 DAP, Koshihikari and GP9-7 canopies covered the soil as shown by their similar and continued low RPPFD values. Tiller emergence of the transplanted weed, *E. crus-galli* var. *formosensis*, one of the most serious weeds in Japan, was significantly suppressed in GP9-7 stand (Figure 5B). The preceding attenuation of incident light under the GP9-7 canopy (Figure 5A) acted to suppress initial weed growth to a level insufficient to increase weed spread. Also, GP9-7 exhibited remarkable weed suppressive activity against the naturally occurring weed, *E. crus-galli* var. *formosensis* (Figure 6). Especially, tiller numbers and dry matter weights of naturally emerging weeds were significantly repressed under GP9-7 canopies (Figures 6B,C). Although GP9-7 did not completely eradicate the weeds in our experiment, the significant repression of weed growth under GP9-7 canopy may reduce nutrients interception by weeds and inhibit weed seed production, two factors that should help relieve farmers of weeding costs. Reduced numbers of herbicide applications will also decrease the environmental load and the risk arising herbicide-resistant weeds (Annett et al., 2014; Heap and Duke, 2018; Islam et al., 2018).

### Yield and Grain Quality of GP9-7

**FIGURE 7:**
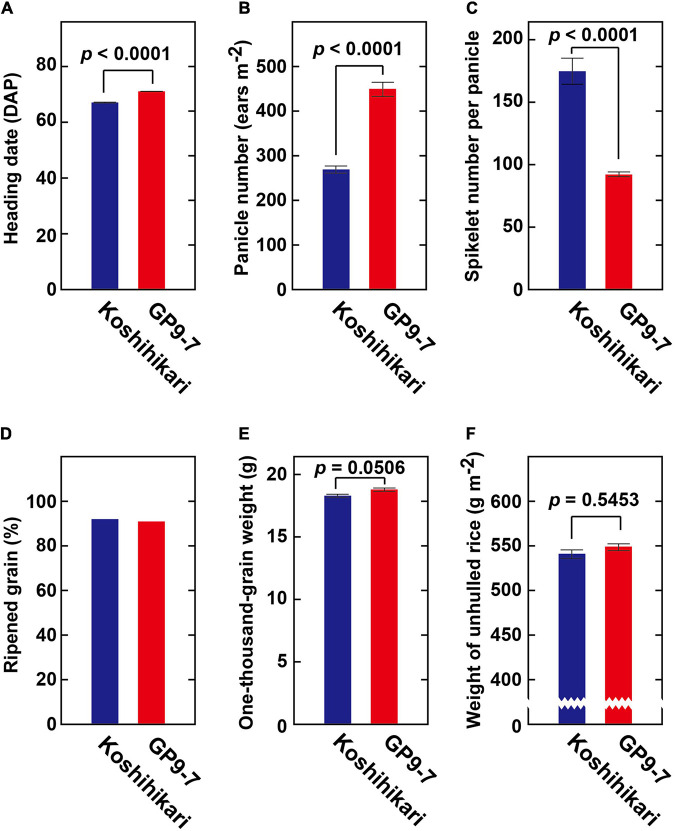
Yield-related traits of GP9-7. **(A)** Heading date (*n* = 12), **(B)** panicle number per m^2^ (*n* = 12), **(C)** spikelet number per panicle (*n* = 12), **(D)** percent ripened grain (*n* = 5), **(E)** one-thousand grain weight (*n* = 3), and **(F)** weight of unhulled rice (*n* = 3) of Koshihikari and GP9-7 cultivated in the test field at Tsukuba, Japan in the summer of 2018 (planting date: May 28, 2018) under sparse conditions (interplant space = 36 cm; 7.7 plants m^–2^). *P*-values were calculated by the Welch’s *t*-test and are reported above the bar graphs. Mean values for Koshihikari and GP9-7 with SE values are indicated with blue and red bars, respectively.

GP9-7 was constructed using the genetic background of Koshihikari, the most widely accepted *japonica* cultivar for staple food in Japan due to its good taste. Since GP9-7 contains a small segment of chromosome 7 from *O. rufipogon* (Figure 1A), GP9-7 displayed slightly delayed heading (Figure 7A), almost twice the number of panicles per m^2^ (Figure 7B), almost half the number of spikelets per panicles (Figure 7C), and a nearly equivalent amount of ripened grain (Figure 7D). The thousand grain weight of GP9-7 was also equivalent to that of Koshihikari (Figure 7E). Consequently, the final yield of GP9-7 was about the same as Koshihikari (Figure 7F). Significant increase of panicle number per plant (Figure 7B) should compensate the panicle number per area, which increase is restricted in existing cultivars under the sparse planting condition. On the other hand, significant promotion of GP9-7 vegetative growth (Figure 3A) did not result in an increase in yield (Figure 7F). We conducted yield tests on the Tsukuba fields from 2016 to 2020 and in the Fukuyama field in 2018 and 2019 (Supplementary Table 3). Despite the cultivation trials over the several summers, no significant increase in yield was detected. Possibly optimal cultivation conditions that bring out the best performance from GP9-7 have not been identified yet. Although the source ability of rice was improved in this study, the sink ability was not manipulated. This possibility may be an alternative reason for why the yield of the NIL did not increase in our experiment. Nevertheless, the introduction of a chromosomal segment that promoted vegetative growth and comparable grain yields is a significant advance since the yields of inbred lines to which a similar chromosomal segment was introduced were reported to be halved (Tan et al., 2008; Hua et al., 2016; Wu et al., 2018). Furthermore, in the yield tests conducted on the Fukuyama fields, no significant reduction of the grain yields of GP9-7 compared with those of Koshihikari was detected even in the semi-dense planting density (Supplementary Table 3), which cultivation condition is close to the previous reports (Tan et al., 2008; Hua et al., 2016; Wu et al., 2018).

A similar inbred line, YIL18, was derived from a cross between the *indica* cultivar, Tequing, as the recipient parent and *O. rufipogon* (accession: YJCWR), as the donor parent (Tan et al., 2008). Another similar inbred line, DIL29, was derived from a cross between the *indica* cultivar, Guichao 2, as the recipient parent and *O. rufipogon* (accession: DXCWR), as the donor parent (Wu et al., 2018). Both the donor accessions, unlike ours, were collected in China. The grain yields of YIL18 and DIL29 were about 57% of Tequing and about 63% of Guichao 2, significantly lead to inferior yields (Tan et al., 2008; Hua et al., 2016; Wu et al., 2018). Hua et al. (2016) examined the canopy structure of YIL18 using three-dimensional digitizing analysis and found a significant decrease in the LAI of YIL18 compared with those of the recurrent parent, Tequing. This report also mentioned that YIL18 had greater mutual shading due to its prostrate plant shape.

We attribute this major discrepancy to three reasons: (1) Inbred lines YIL18 and DIL29 contain several wild rice chromosome segments in addition to the chromosome 7 segment. These additional chromosome segments may be responsible for the yield reduction; (2) In these cases, the recurrent parents were the *indica* cultivars, whereas we used Koshihikari, a *japonica* cultivar; and (3) The chromosomal donor in this study was collected from Thailand, whereas the donors in previous studies were collected from China. It is possible that differences in the sequence of this chromosomal segment may impart different traits. In fact, the *Prog1* sequence of GP9-7 was different from those of accessions YJCWR and DXCWR (Supplementary Figure 3B). Progress in whole-genome sequence analysis of these accessions may shed light on the reasons for these discrepancies. Identifying which of the three types of *O. rufipogon* (Or-I, -II, and -III) (Huang et al., 2012) by genome-wide association analyses that accurately describe these accessions may sort out these confusing results.

**FIGURE 8:**
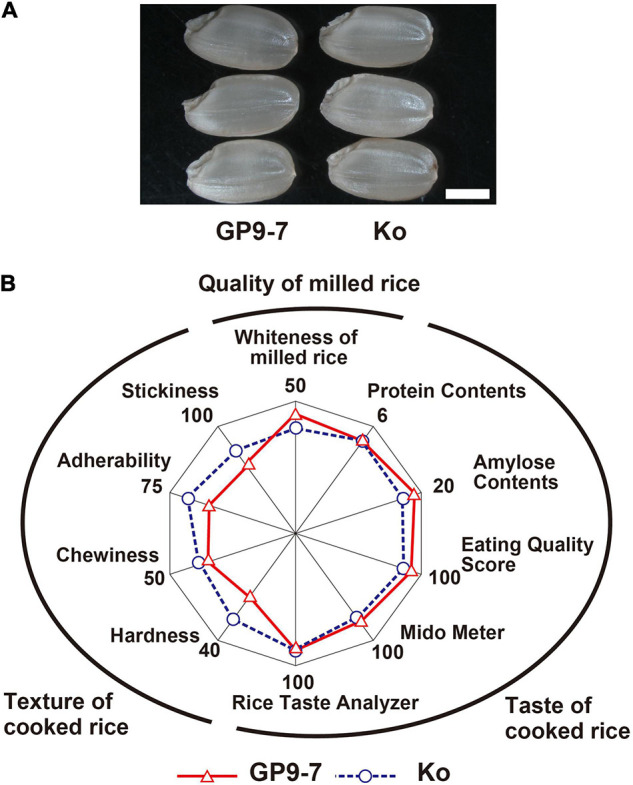
Grain quality-related traits of GP9-7 harvested in the Tsukuba field in the fall of 2018. **(A)** A morphological comparison of typical hulled rice grains of Koshihikari and GP9-7. Scale bar: 2 mm. **(B)** Summary of rice texture and taste quality tests. Values for Koshihikari are indicated with blue circles with broken lines and values for GP9-7 are represented with red triangles with solid lines.

The surface of GP9-7 milled grains was the white-like Koshihikari (Figure 8). The taste-related factors of GP9-7 were nearly identical except for the amylose content that was very slightly higher in GP9-7 compared to Koshihikari (Figure 8B). Cooked rice from GP9-7 had relatively softer texture factors (Figure 8B), suggesting that the introduced chromosomal segment of wild rice in this study did not negatively influence grain quality, including its taste.

The remaining flaw of GP9-7 was lowered lodging resistance attributed to the smaller stem diameters that are a trade-off for the increased number of tillers. We believe that improvements such as the introduction of alleles conferring lodging resistance are possible. Lodging resistance loci have been well studied and exploitation of them will overcome this flaw (Shah et al., 2019).

### Influences of This Research on the Future

This study attained that construction of the NIL capable of accelerating vegetative growth and suppressing weeds using wild rice genetic resources without undesirable consequences and yield reduction. The practical use of the NIL or the chromosomal segment that causes these traits may reduce weed control costs and environmental consequences associated with herbicide application, which open a way to solve some obstacles to the United Nations SDGs. This study also indicates that the genetic diversity of wild rice deserted in the domestication process is a valuable resource for breeding new cultivars with desired traits. The Japanese concept of *MOTTAINAI* has to be pushed to the front in this case; we have an obligation to excavate and apply these precious resources hidden in wild relatives in order to realize a bright future.

## Data Availability Statement

The datasets presented in this study can be found in online repositories. The names of the repository/repositories and accession number(s) can be found below: GenBank/EMBL/DDBJ Acc. Nos. LC573903 and LC573904.

## Author Contributions

NI, HA, and KI designed the research and performed the experiments. HH contributed to the construction of the NIL. AU and TI provided critical supports for assaying weed control. NI and HA wrote most of the manuscript with help from all the other authors. All authors read and approved the manuscript.

## Conflict of Interest

The authors declare that the research was conducted in the absence of any commercial or financial relationships that could be construed as a potential conflict of interest.

## Publisher’s Note

All claims expressed in this article are solely those of the authors and do not necessarily represent those of their affiliated organizations, or those of the publisher, the editors and the reviewers. Any product that may be evaluated in this article, or claim that may be made by its manufacturer, is not guaranteed or endorsed by the publisher.

## Footnotes

1
https://sdgs.un.org

2
https://imagej.nih.gov/ij/

3
https://www.r-project.org
