# Supplementary material for: A Rice Ancestral Genetic Resource Conferring Ideal Plant Shapes for Vegetative Growth and Weed Suppression
Source: Front Plant Sci. 2021 Nov 26;12:748531. doi: 10.3389/fpls.2021.748531 (PMC8664436; doi:10.3389/fpls.2021.748531)
Supplement: Supplementary file 1 [file Data_Sheet_1.docx]

Supplementary Material


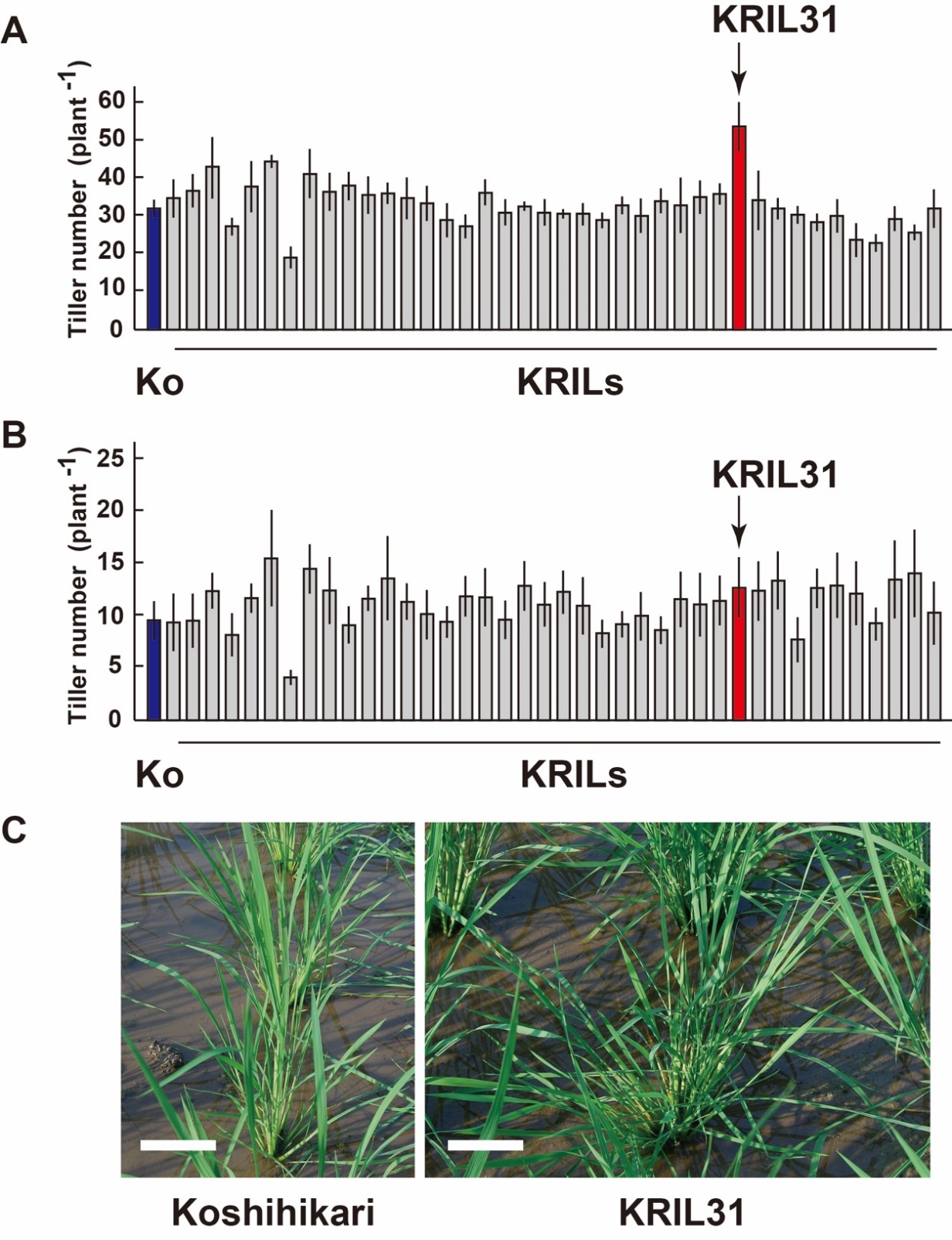


**Supplementary Figure 1.** **Properties of KRIL31, the chromosome donor for GP9-7.**

Tiller numbers of each line from the KRIL chromosome segment substitution lines (Hirabayashi et al., 2010) at the ripening stage grown under sparse (A) and dense (B) planting densities. The means of 5 plants and their SD values are shown. Koshihikari (Ko: blue bar) and KRILs were cultivated in a test field at Tsukuba, Japan in the summer of 2010. The number of tillers of KRIL31 are indicated by red bars. Photographs C document the typical plant shapes of Koshihikari and KRIL31 under the sparse planting conditions. These photographs were taken at 29 DAP. Scale bars: 10 cm.


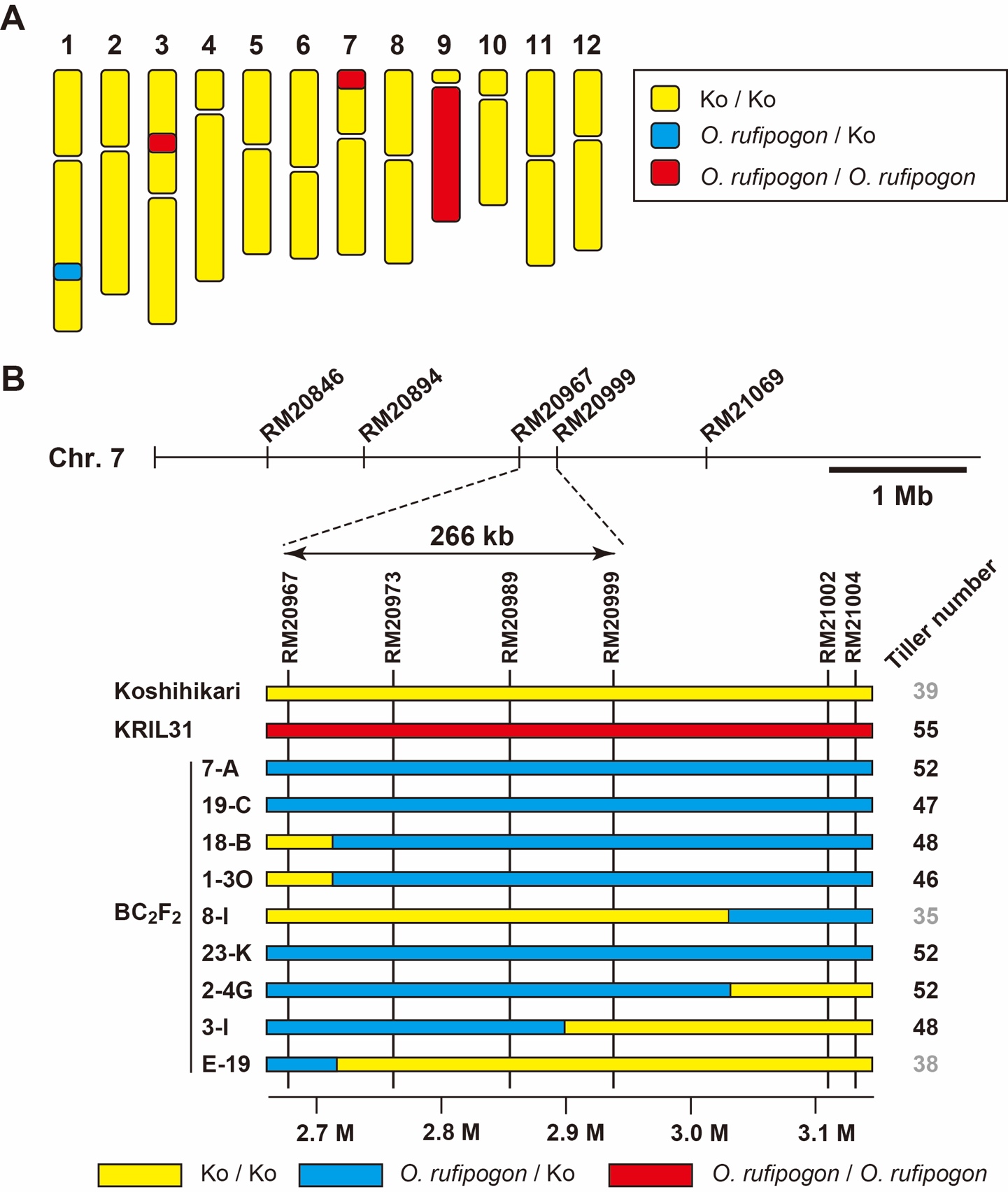


**Supplementary Figure 2.** **Schematic genotype of KRIL31 and genotypic-phenotypic analyses to detect the corresponding segment controlling tiller spreading and increase.**

Graphic representation of the KRIL31 genotype (A) (Hirabayashi et al., 2010). Diagram B summarizes the genotypic-phenotypic analyses of plants from the BC_2_F_2_ generation to locate the corresponding segment controlling tiller spreading and increase in tiller number relative to SSR markers. The yellow zones indicate the segments derived from Koshihikari, and the red zones represent the segments contributed by *O. rufipogon*. Blue zones show the heterozygous segments of both genotypes.


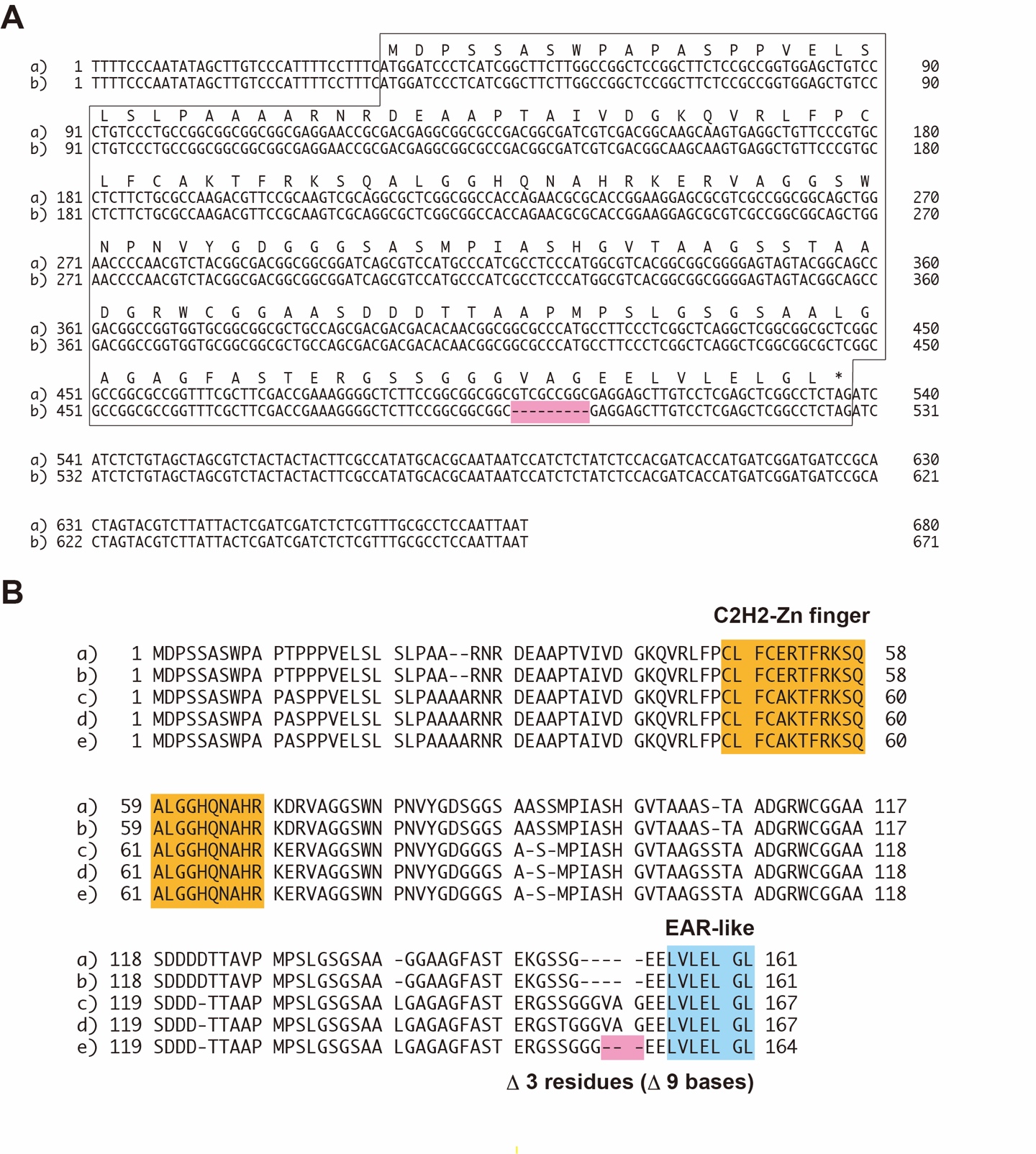


**Supplementary Figure 3.** **Comparison of sequences of the Prog1 from Koshihikari, several accessions of *O. rufipogon* and GP9-7.**

(A) Comparison of nucleotide sequences of the *Prog1* gene from Koshihikari and GP9-7. Sequence a: Koshihikari (DDBJ/EMBL/GenBank: LC573903), sequence b: GP9-7 (DDBJ/EMBL/GenBank: LC573904). (B) Comparison of the deduced amino acid sequences of Prog1 from several accessions of *O. rufipogon*, Koshihikari and GP9-7. Sequence a: *O. rufipogon* (accession: YJCWR; GenBank: ACE06775.1), sequence b: *O. rufipogon* (isolate: YD2-0772; GenBank: ACE06777.1), Sequence c: Koshihikari, sequence d: *O. rufipogon* (accession: DXCWR; GenBank: ASR75314.1) and sequence e: GP9-7. The deletion detected in GP9-7 is highlighted with a pink background. Characteristic motifs shown in Prog1, such as the C_2_H_2_-type Zn-finger domains (Agarwal et al., 2007) and the EAR-like motif (Kagale et al., 2010) are highlighted with orange and blue backgrounds, respectively.


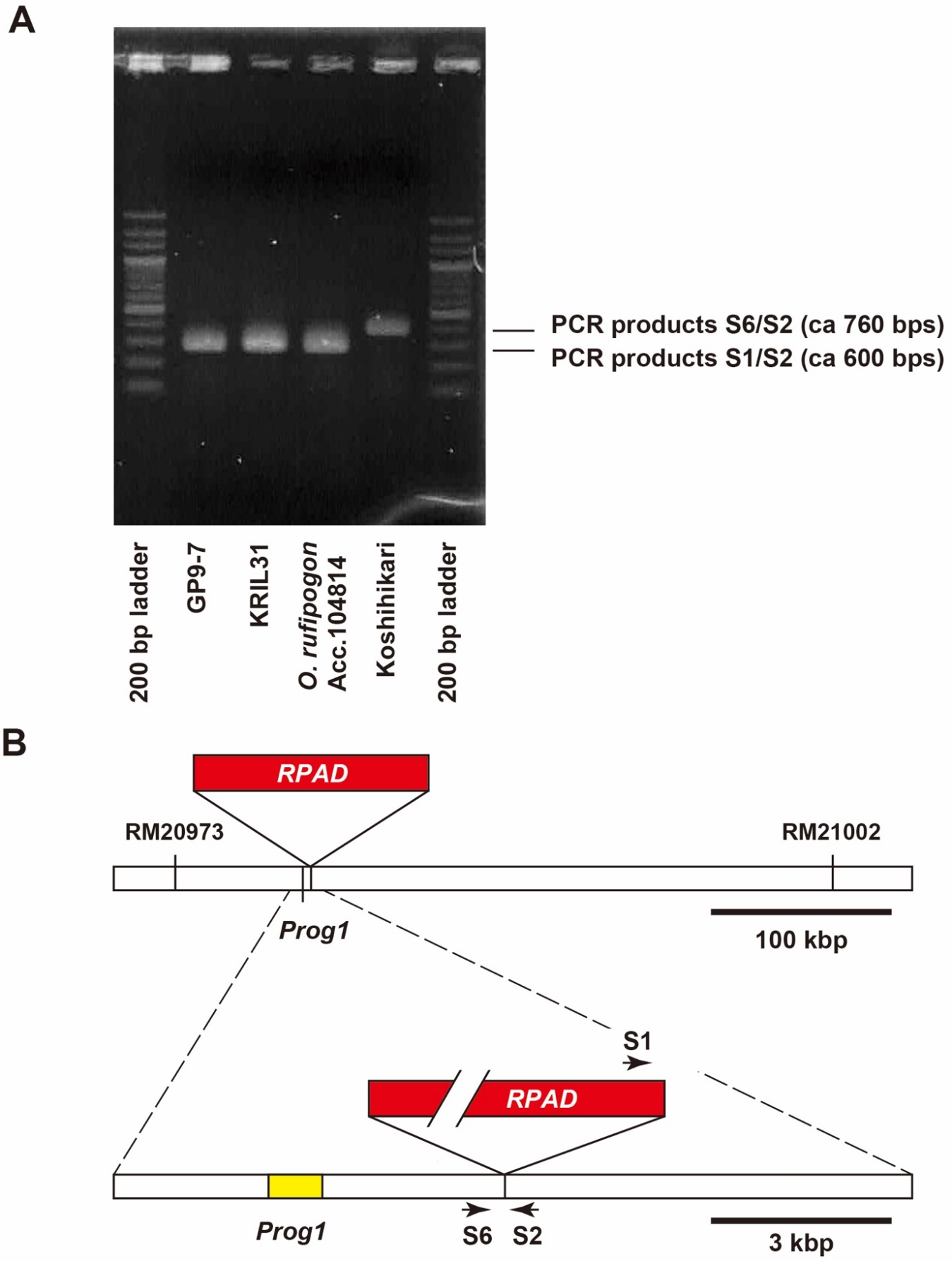


**Supplementary Figure 4.** **Verification of the *RPAD* insertion in GP9-7 by PCR analysis.**

(A) Verification of *RPAD* insertion (Wu et al., 2018) by agarose gel electrophoresis of PCR products. The diagram B summarizes PCR primer design and positional relationships among *Prog1*, the SSR markers and *RPAD*. The upper bar represents ca. 440 kbp segment of chromosome 7 located between two SSR markers, RM20973 and RM21002. The lower bar represents a magnified 13.5 kbp region of chromosome 7 showing the location used for PCR primer design (arrows).


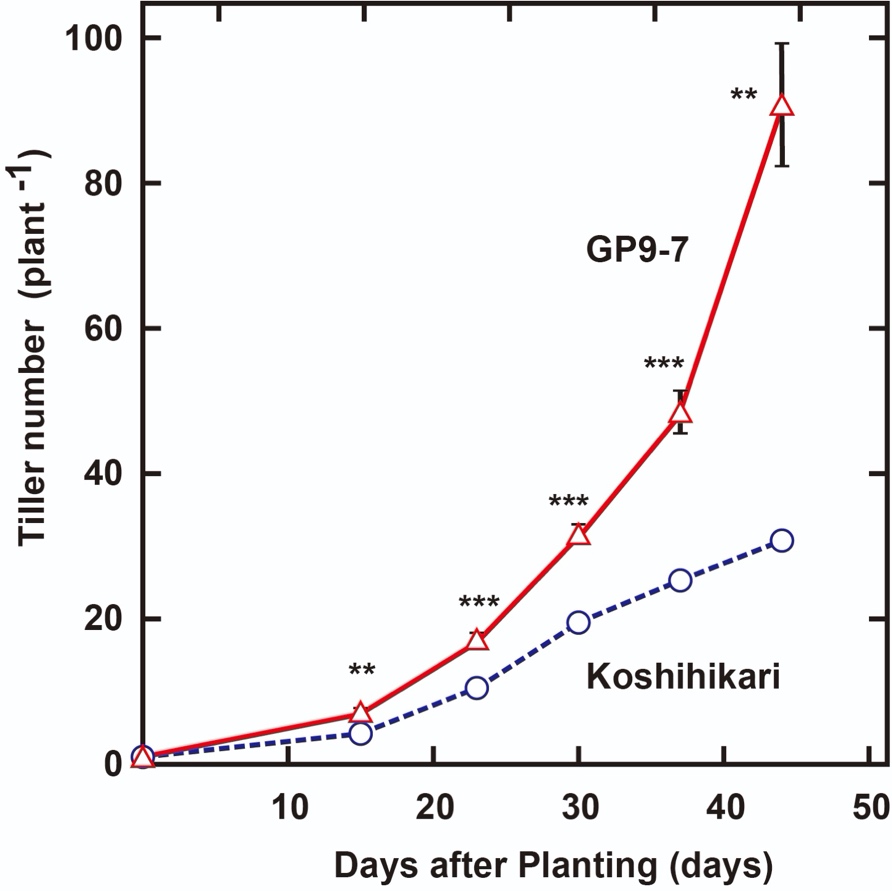


**Supplementary Figure 5.** **Increase in tiller numbers of Koshihikari and GP9-7 during the early vegetative growth phase.**

Increase in tiller numbers of Koshihikari (blue circles with broken lines) and GP9-7 (red triangles with solid lines) during the early vegetative growth phase in the test field in Tsukuba, Japan. Mean values of 6 plants with SE values are plotted. Symbols, ***, ** and * indicate statistically significant differences compared to Koshihikari at *p* < 0.001, *p* < 0.01 and *p* < 0.05, respectively, calculated by Welch’s *t*-test.


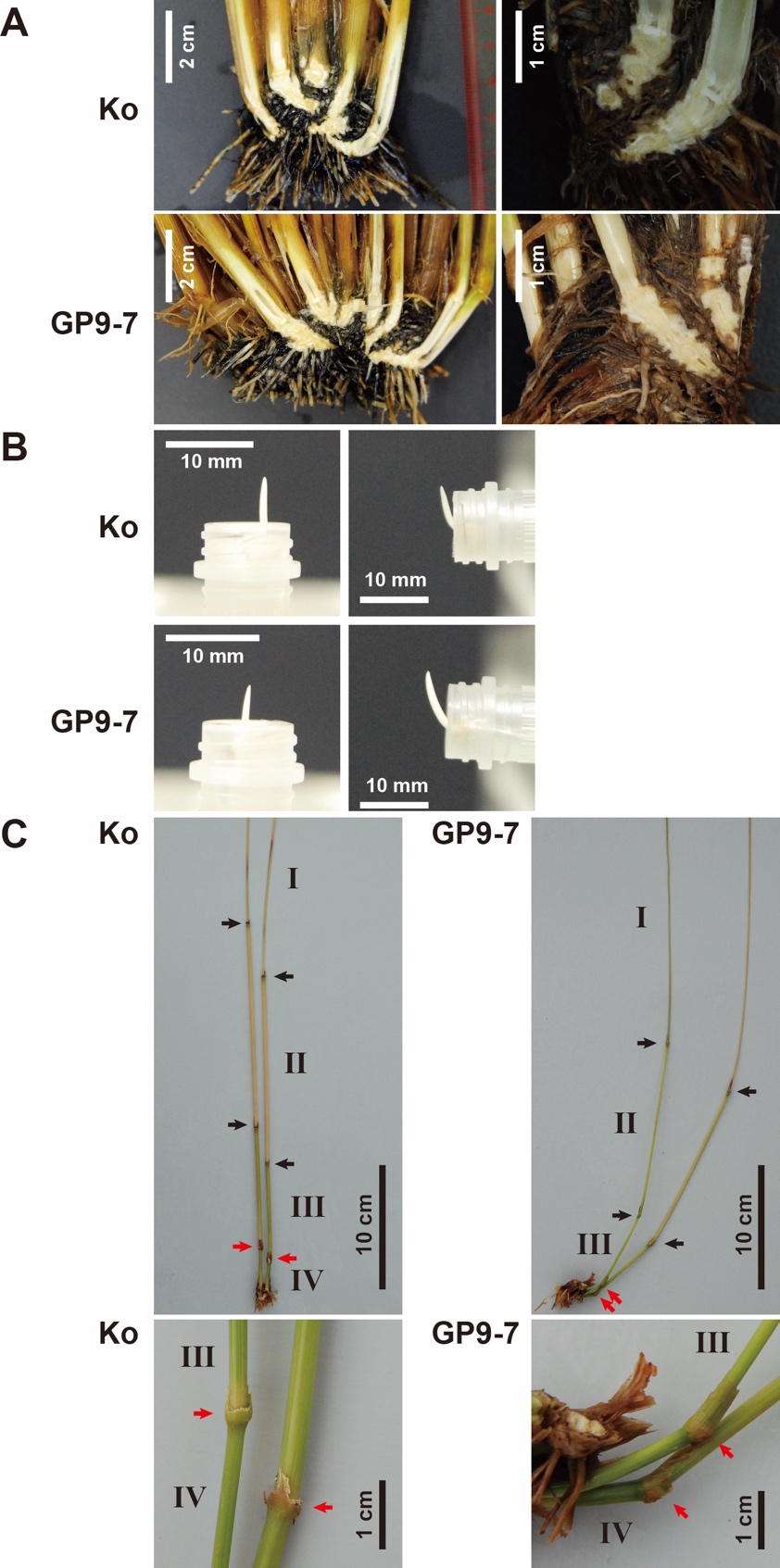


**Supplementary Figure 6.** **Photographs showing the tiller bases and the gravitropic responses of Koshihikari and GP9-7.**

Photographs A showing the cut surfaces of the tiller bases of Koshihikari and the GP9-7 at the fully ripe stage. Photographs B indicating the gravitropic responses of coleoptiles of Koshihikari and GP9-7. Photographs on the right show the gravitropic response of the seedlings leaned horizontally at the midpoint of the incubation. Photographs C displaying the gravitropic bend at nodes between the elongated internodes of Koshihikari and GP9-7 at the fully ripe stage. All leaf sheaths were removed manually. Arrows point to node positions. In particular, node positions between internode III and IV are indicated by red arrows. Roman numerals identify internode numbers.

**Supplementary Table 1. A summary of the climate conditions of the fields used in this study.**

**
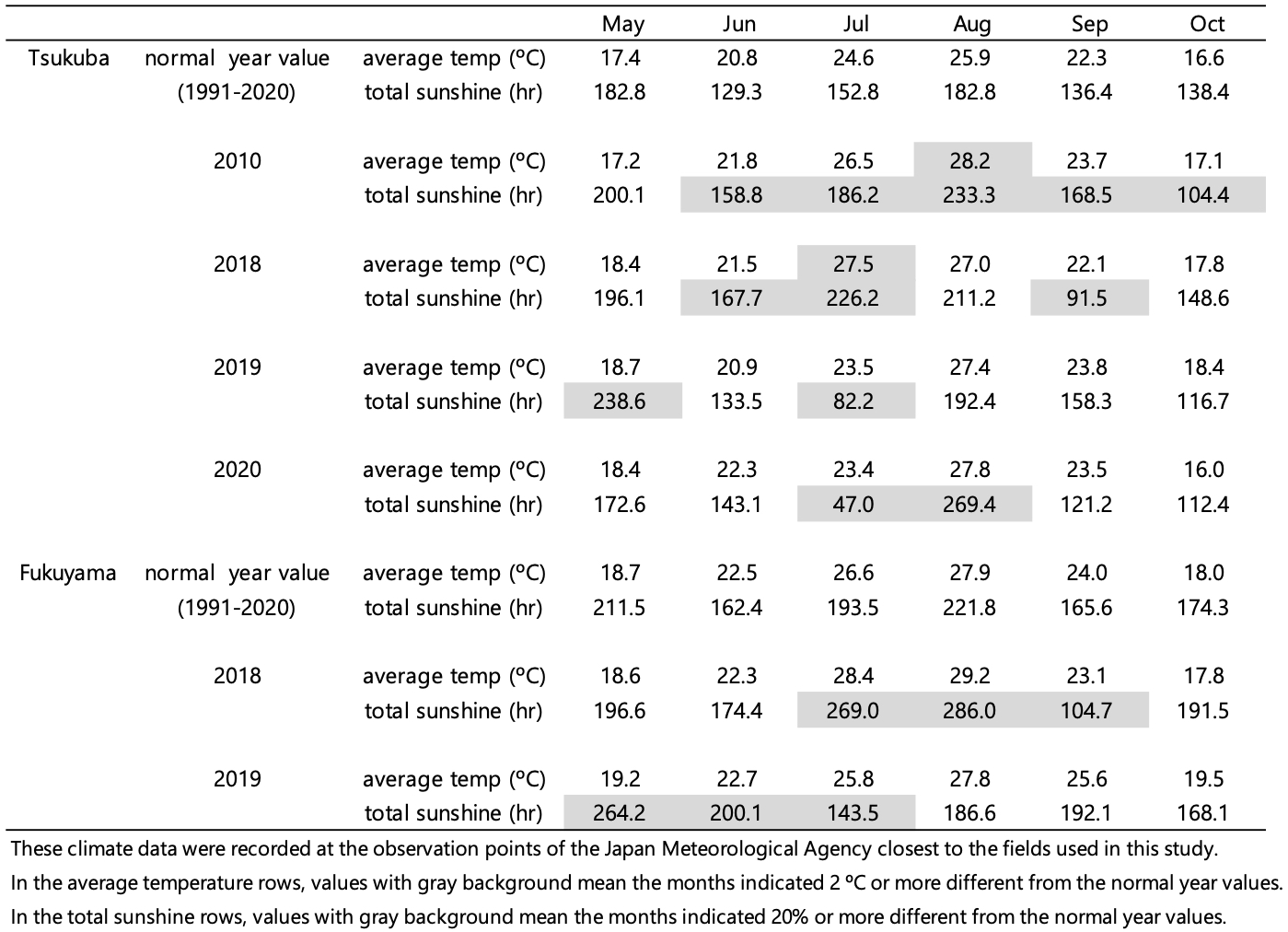
**

**Supplementary Table 2. Sequences of PCR primers used in this study.**

**
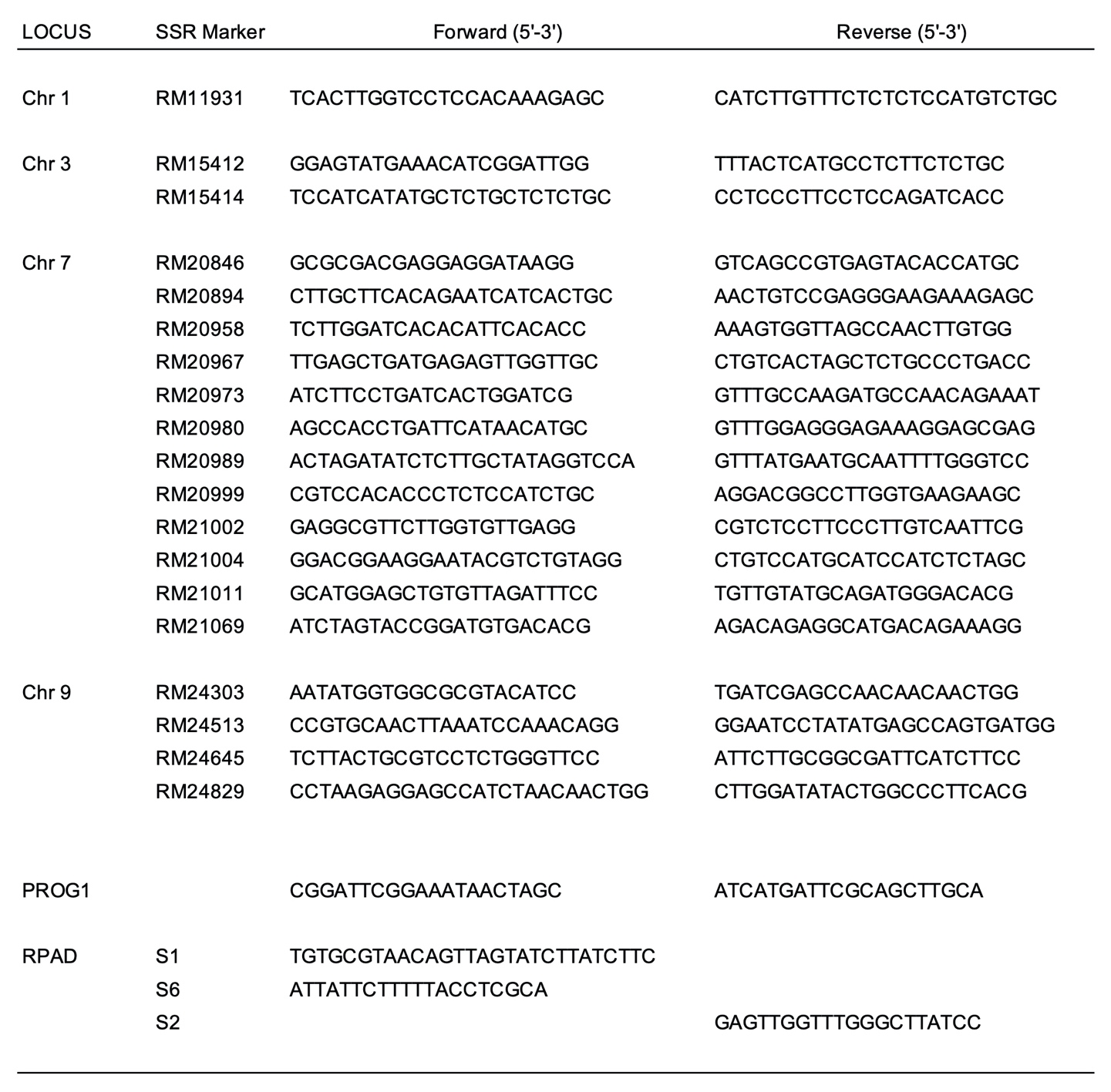
**

**Supplementary Table 3. A summary of yield survey data conducted for multiple years.**

**
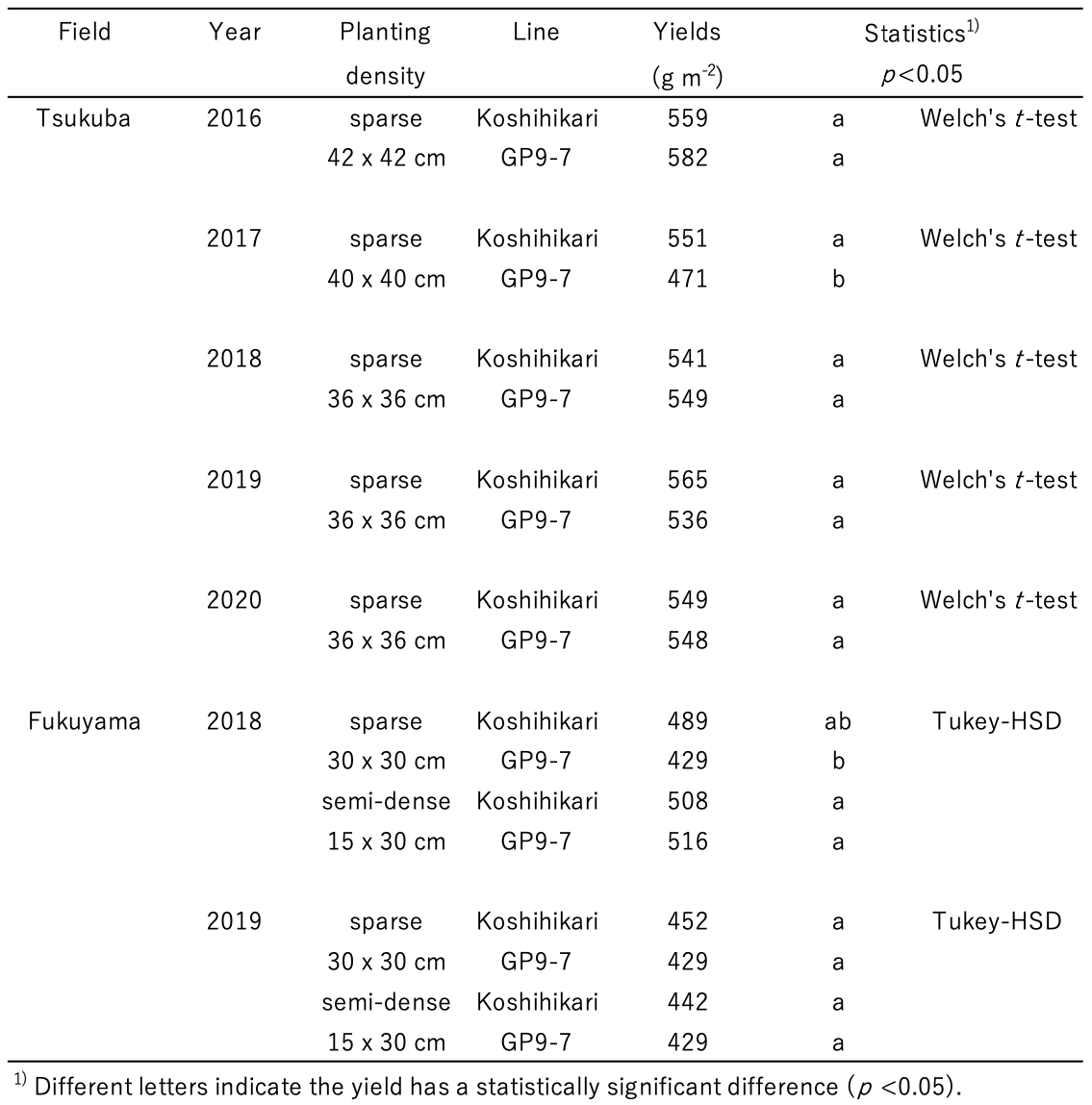
**

**Supplementary Movie 1. Tiller spreading during the early vegetative growth phase of Koshihikari (left) and GP9-7 (right).**

For the video, 31 days was compressed into 8 seconds. The black triangles on the backboard are spaced 10 cm apart.

**Supplementary Movie 2. Tiller closing during the transition from the vegetative to the reproductive growth phases of Koshihikari (left) and GP9-7 (right).**

For the video, 39 days was compressed into 10 seconds. The black triangles on the backboard are spaced 10 cm apart.

**References**

Agarwal, P., Arora, R., Ray, S., Singh, A.K., Singh, V.P., Takatsuji, H., et al. (2007). Genome-wide identification of C_2_H_2_ zinc-finger gene family in rice and their phylogeny and expression analysis. *Plant Mol. Biol.* 65**,** 467-485. doi: 10.1007/s11103-007-9199-y.

Hirabayashi, H., Sato, H., Nonoue, Y., Kuno-Takemoto, Y., Takeuchi, Y., Kato, H., et al. (2010). Development of introgression lines derived from *Oryza rufipogon* and *O. glumaepatula* in the genetic background of *japonica* cultivated rice (*O. sativa* L.) and evaluation of resistance to rice blast. *Breed. Sci.* 60**,** 604-612. doi: 10.1270/jsbbs.60.604.

Kagale, S., Links, M.G., and Rozwadowski, K. (2010). Genome-wide analysis of ethylene-responsive element binding factor-associated amphiphilic repression motif-containing transcriptional regulators in Arabidopsis. *Plant Physiol.* 152**,** 1109-1134. doi: 10.1104/pp.109.151704.

Wu, Y., Zhao, S., Li, X., Zhang, B., Jiang, L., Tang, Y., et al. (2018). Deletions linked to *PROG1* gene participate in plant architecture domestication in Asian and African rice. *Nat. Commun.* 9**,** 4157. doi: 10.1038/s41467-018-06509-2.
